# From First Slip to Second Setback: Intracranial Injury Risk after Recurrent Falls in Older Adults: A Retrospective Cohort Study

**DOI:** 10.20900/agmr20250012

**Published:** 2025-06-23

**Authors:** Asmaa Namoos, Nicholas Thomson Thomson, Tarek Zaho, Amanda Rudderman, Michel Aboutanos

**Affiliations:** The Injury and Violence Prevention Program, Departments of Surgery, Virginia Commonwealth University, Richmond, VA 23284, USA

**Keywords:** falls, older adults, recurrent falls, intracranial injuries (ICIs), fall prevention, risk factors

## Abstract

**Background:**

Recurrent falls in older adults are a major public health concern, often resulting in severe injuries such as Intracranial Injuries (ICIs). These injuries increase the risk of cognitive decline, disability, and early mortality. While medications like antihypertensives and antidepressants are essential for managing chronic conditions, they may also contribute to fall-related injury risk.

**Methods:**

This retrospective cohort study analyzed data from 3760 adults aged 65 to 89 years at the Virginia Commonwealth University Health System (VCUHS) in 2023. Patients were categorized into two cohorts: those with initial falls (*n* = 2710) and those with recurrent falls (*n* = 1050). Demographic variables, medication use, and fall-related ICI outcomes were examined. Incidence, prevalence, and measures of association were calculated. Cox regression models assessed the impact of demographic and clinical predictors on ICI risk.

**Results:**

The risk of ICI was 10.4% higher in patients with recurrent falls compared to those with initial falls (HR = 1.104, 95% CI: 0.833–1.463, *p* = 0.0493). Males were significantly more likely to sustain ICIs (HR = 1.369, 95% CI: 1.054–1.779, *p* = 0.0186), as were older adults with each additional year of age (HR = 1.021, 95% CI: 1.001–1.041, *p* = 0.0347). Antihypertensive use nearly doubled the risk of ICI (HR = 1.958, 95% CI: 1.379–2.781, *p* = 0.0002), and antidepressant use was associated with a 47.1% increase (HR = 1.471, 95% CI: 1.1–1.968, *p* = 0.0093). Race was not a significant predictor of ICI risk in adjusted models.

**Conclusions:**

Recurrent falls were associated with a higher likelihood of ICIs among older adults, particularly in males, those of advanced age, and individuals prescribed antihypertensive or antidepressant medications. These results point to the importance of identifying patients at increased vulnerability and tailoring fall-related care and medication reviews accordingly to help reduce injury severity in this population.

## BACKGROUND

Falls are among the most frequent and severe health incidents affecting older adults, with millions of individuals over the age of 65 experiencing a fall each year [1]. For many, an initial fall not only represents a physical injury but also serves as a predictor for future falls, marking the beginning of a potentially dangerous cycle [2]. Research shows that an initial fall frequently leads to reduced physical activity due to a newfound fear of falling, which, in turn, results in decreased muscle strength, balance, and flexibility [3,4]. This cycle of physical deconditioning can increase vulnerability to future falls, creating a compounding effect where each fall raises the likelihood and severity of the next. Recurrent falls, those experienced by individuals who fall multiple times within a short period, are a particularly alarming phenomenon [5]. Studies have shown that the likelihood of a second fall can increase substantially following the initial [6], placing older adults at a high risk of severe health consequences and signaling a potential decline in mobility, independence, and overall health [7]. Recurrent falls often occur within a year of the initial fall and are commonly associated with a higher degree of injury, leading to prolonged recovery times, frequent hospitalizations, and a reduced quality of life [8,9].

Additionally, certain medications are known to contribute to fall risk in older adults. Antihypertensive medications, which help manage high blood pressure, can sometimes lead to dizziness and orthostatic hypotension, increasing the risk of falls [10]. Similarly, antidepressants, particularly those affecting balance and alertness, may also contribute to instability and heighten fall risk among older adults [11]. The combined effects of these medications can be particularly challenging for individuals with a history of falls, as their impact on balance and blood pressure may exacerbate the cycle of recurrent falls and associated injuries [12].

With each additional fall, the risk of more serious health outcomes escalates, as repeated trauma increases the likelihood of significant injuries and prolonged recovery times. Recurrent falls are not only more frequent but also tend to involve more severe injuries, such as fractures or ICIs, which can further impair mobility and functional independence [13]. ICIs, which include traumatic brain injuries (TBIs) and concussions, are especially hazardous in older adults due to the fragility of brain structures and decreased physiological resilience with age [14,15]. Following even a mild head impact, older individuals may experience complications that can lead to lasting cognitive impairment, disability, or death [16]. Research highlights that older adults with a history of falls have a heightened susceptibility to ICIs with each subsequent fall, as weakened balance, slowed reaction times, and pre-existing health conditions exacerbate the impact of repeated trauma to the head [17].

Given the serious implications of ICIs following recurrent falls, understanding the specific factors that contribute to these risks is essential. Prior studies have shown that demographic characteristics (such as age, sex, and race) and the timing of follow-up after an initial fall can significantly influence injury outcomes and survival rates [18]. However, there is a gap in the literature regarding the longitudinal impact of recurrent falls on survival, particularly in the context of ICIs among older adults. By exploring these factors, our study seeks to address this gap and build a framework for tailored community-based interventions aimed at reducing the recurrence of severe fall-related injuries.

The purpose of this study is to analyze the demographic characteristics, incidence and prevalence, risk associations with initial and recurrent falls resulting in ICIs among older adults. By examining factors such as sex, race, gender and medication, this study aims to provide a comprehensive understanding of the risks and outcomes associated with fall recurrence.

## METHODS

### Study Design

A retrospective cohort study focused on older adults, aged 65 to 89 years old, who experienced either initial or recurrent falls resulting in ICIs between Jan to December 2023. Participants were categorized into two distinct cohorts based on their fall history within this period. The “Initial Falls” cohort included 2710 patients who encountered their initial fall incident, while the “Recurrent Falls” cohort comprised 1050 patients who had multiple fall incidents within the same timeframe.

### Data Source

TriNetX is an extensive health research network that links healthcare organizations with life sciences companies, offering real-world data to support clinical research and the discovery of new treatments. The platform grants researchers access to anonymized and aggregated patient data from electronic health records (EHRs), facilitating observational studies, optimizing clinical trials, and generating evidence-based insights to drive advancements in healthcare [19].

### Data Collection

Data were extracted from the Electronic Health Record (EHRs) available through the TriNetX network at Virginia Commonwealth University (VCUHS). The dataset includes detailed demographic information, such as age, sex, race, and ethnicity, along with ICD-10 diagnosis codes [20]. Additionally, it records information on falls, recurrence of falls, types of ICIs, and specific injury types associated with each fall incident. The dataset also captures the timing and severity of falls, hospital visits related to these incidents, and clinical history relevant to fall risk factors. This comprehensive dataset allows for an in-depth analysis of the relationships between comorbidities, demographic factors, and fall risk in an elderly population.

### Patient Selection

Patients were categorized into two cohorts, those experiencing an initial fall and those with recurrent falls within one year. We included patients aged 65 to 89 who experienced at least one fall and had a documented ICI within the year 2023. Patients without an ICI diagnosis were excluded from the final analysis. Individuals were categorized into two cohorts: those who experienced an initial fall with an ICI, and those with recurrent falls within the same year who also sustained an ICI. Due to limitations in the TriNetX data structure, we could not capture the exact number or timing of multiple recurrent falls beyond the study window. Therefore, patients with multiple fall incidents during 2023 were collapsed into a single outcome per individual, based on whether a recurrence occurred during that year. Specifically, Cohort 1 comprised patients who experienced their initial fall, while Cohort 2 included those who had multiple fall incidents within the same timeframe. To ensure relevance to the study’s aims, we further filtered the dataset to include only those patients who sustained ICIs within one year following either their initial fall or recurrent falls. For this study, ICIs were defined as a spectrum of head injuries resulting from external trauma, including cerebral hemorrhage, subarachnoid hematoma, concussion, traumatic brain injury, and ischemic lesions. While loss of consciousness was captured through specific ICD-10 codes, it was not the sole criterion for classification. The diagnostic criteria were based on the following ICD-10 codes related to falls and ICIs (Table 1).

We utilized the RxNorm coding system to classify medication usage among older adults, specifically focusing on those medications associated with an increased risk of falls. We identified antihypertensive medications using the RxNorm code ‘CV490’ and antidepressants using the RxNorm code ‘N06A’ (Table 2).

### Handling of Multiple Records and Missing Data

For this study, each patient was treated as a unique unit of analysis. In instances where multiple fall incidents were recorded for the same patient within the study period, only the initial fall incident was included in the analysis. Subsequent fall incidents occurring more than one year after the initial fall were recorded to capture recurrent falls but were not treated as separate events for the purpose of this study.

The TriNetX platform ensures high standards of data quality and completeness. Consequently, the dataset utilized in this study had no missing values for key variables, including diagnosis codes and demographic information, allowing for comprehensive analysis without the need for data imputation [19].

### For Missing Data

The TriNetX platform aggregates real-world data from EHRs across healthcare organizations, ensuring that all necessary patient data are complete and de-identified. TriNetX’s federated data network maintains rigorous data governance protocols, minimizing the occurrence of missing data. Therefore, for this analysis, there were no missing data for key variables such as diagnosis codes, demographic characteristics, or clinical outcomes.

### Incidence and Prevalence Calculation

In TriNetX, the incidence proportion refers to the number of new cases of a particular condition that arise in a specific group of patients during a set time frame. To be counted, patients must have their medical records overlapping with this time period and should not have been diagnosed with the condition before that. The incidence rate considers how long patients were at risk, which is determined by multiplying the number of patients by the number of days in the time frame. This way, both the number of new cases and the total number of patients considered are consistent with what we use to calculate the incidence proportion.

On the other hand, prevalence captures all existing cases of the condition at any point before or during the time window. The denominator for prevalence includes all patients whose records overlap with the time period, while the numerator consists of those who were diagnosed with the condition either prior to or within that time frame. These methods adhere to the established procedures for analyzing real-world data from EHR in TriNetX [21].

### Statistical Analysis

The statistical analysis was performed using the TriNetX platform. Descriptive statistics summarized the demographic characteristics of the study population, with continuous variables (such as age) presented as means and standard deviations, and categorical variables (such as sex, race, and ethnicity) shown as frequencies and percentages. Univariate analyses were conducted using Chi-square tests for categorical variables and t-tests for continuous variables to compare the two cohorts (initial and recurrent falls).

Measures of association between fall recurrence and ICIs were further analyzed through risk ratios and odds ratios, providing insight into the likelihood of ICIs occurring in recurrent fall cases versus initial fall cases.

### Software and Tools

All analyses were conducted using the TriNetX platform’s analytical tools. These tools enabled processing of large datasets and provided robust statistical evaluations and visualizations to support epidemiological analysis.

## RESULTS

### Demographic Characteristic

**Figure 1.**
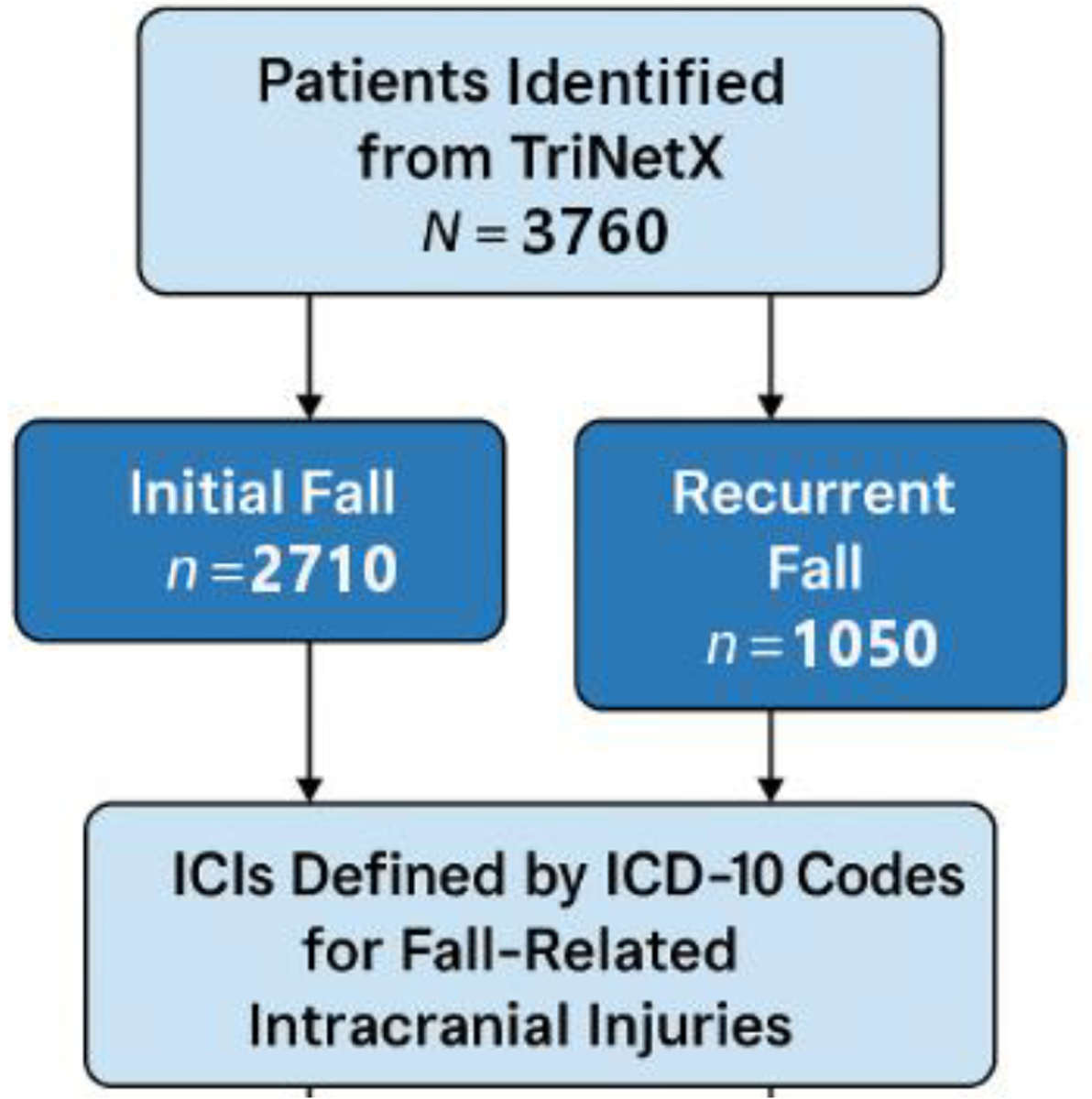
Study flow chart of patient selection, cohort grouping (initial vs. recurrent falls).

The comparative analysis of the demographics and baseline characteristics for older adults aged 65 to 89, who experienced initial or recurrent falls resulting in ICIs in 2023, provides insightful distinctions between two patient cohorts. Cohort 1, comprising 2710 patients who experienced their first fall incident, and Cohort 2, consisting of 1050 patients with recurrent falls, were analyzed for differences in age, sex, and race. Both cohorts exhibit similar age distributions with Cohort 1 having a mean age of 76.1 years (SD = 6.87) and Cohort 2 having a mean age of 76 years (SD = 6.83). The associated *p*-value of 0.7023 suggests no significant difference in age between those experiencing initial and recurrent falls (Figure 1).

There are no significant differences in sex distributions, Cohort 1 included 1580 females and 1140 males, while Cohort 2 comprised 590 females and 470 males. The *p*-values for females and males are 0.2395 and 0.1340, respectively.

Racial composition presented notable findings. While the majority of both cohorts were White (64% in Cohort 1 and 68% in Cohort 2), the difference was statistically significant with a *p*-value of 0.0488, suggesting a disparity in the recurrence of falls among White individuals. For Black or African American participants, no significant difference was found (31% in Cohort 1 vs. 28% in Cohort 2, *p*-value = 0.0704). A striking difference was observed in the proportion of Native Hawaiian or Other Pacific Islander individuals between the cohorts; less than 1% in Cohort 1 versus 1% in Cohort 2, with a highly significant *p*-value of less than 0.0001, which might indicate specific risk factors affecting this group (Table 3).

### Incidence and Prevalences

In Table 4, we present the incidence proportions, prevalence, and incidence rates (cases/person-day) of ICIs following falls across various demographic categories. The analysis reveals that among older adults aged 65 and older, the incidence proportion of ICIs ranges from 0.068 in the 70 to 74 age group to 0.125 for those aged 85 and older, with a corresponding prevalence of 0.127 and 0.154, respectively. The incidence rates also show variability, with the highest rate of 0.000477 observed in the 85 and older category.

Sex-based analysis indicates that males experience a higher incidence proportion (0.086) and prevalence (0.149) of ICIs compared to females, who have proportions of 0.068 and 0.127, respectively. The category for unknown gender shows no applicable data.

When examining racial demographics, a noteworthy finding is that American Indian or Alaska Native individuals show an incidence proportion and prevalence of 1.000, suggesting a potential data anomaly or unique risk profile within this group. Other races, such as Asian and Black or African American, present lower incidence proportions of 0.500 and 0.066, respectively. Notably, the data for Native Hawaiian or Other Pacific Islander is missing, indicating gaps in reporting for this category. White individuals have an incidence proportion of 0.080 and prevalence of 0.138, while those identified as Other Race show a higher incidence proportion of 0.200.

**Figure 2.**
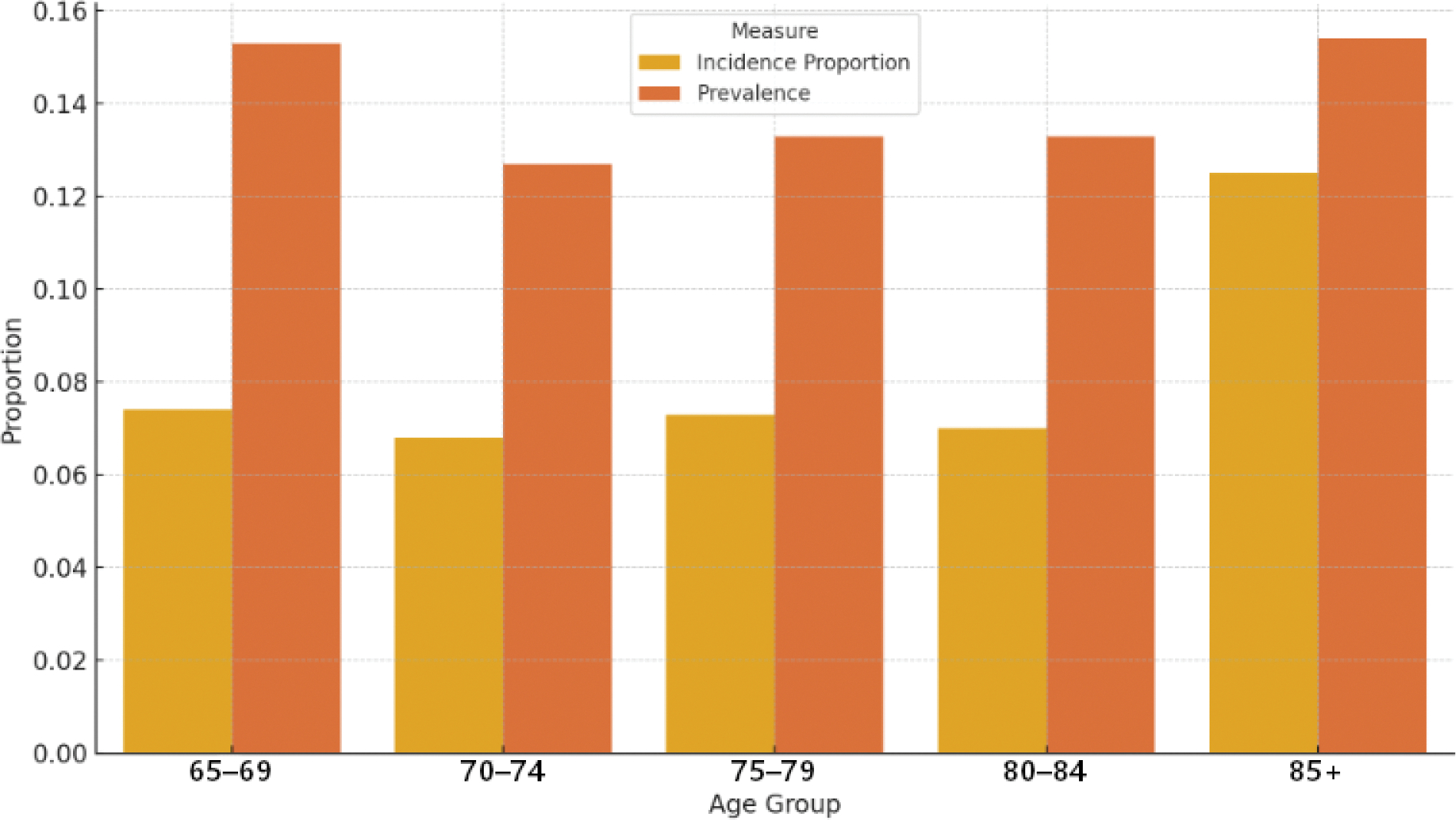
Incidence and Prevalence of ICIs by age group.

Table 5 offers a look at the incidence and prevalence of recurrent falls and the related ICIs among older adults. The data shows that those aged 85 and older face a significant challenge, with a high incidence proportion of 0.222 and a prevalence of 0.300. This highlights how vulnerable this age group is when it comes to recurrent falls. For younger groups, like those aged 65 to 69, the incidence proportion is lower at 0.100, but it’s still a notable concern, with a prevalence of 0.217 (Figure 2).

When we break it down by sex, males seem to have a slightly higher incidence proportion (0.095) and prevalence (0.170) of recurrent falls compared to females, who have figures of 0.075 and 0.153, respectively. Interestingly, there’s no data for individuals with unknown gender, which suggests we might need to improve our data collection in that area.

Looking at race, the numbers tell a mixed story. There are no reported incidences for American Indian or Alaska Native and Asian groups, which might indicate they have very low rates or that we’re missing some data. However, Black or African American individuals show an incidence proportion of 0.080 and a prevalence of 0.172, while White individuals report slightly lower figures at 0.077 for incidence and 0.155 for prevalence. The unknown race category stands out with a high incidence proportion of 0.333, which raises some questions about the experiences of this group. Lastly, those identified as Other Race show an even higher incidence proportion of 0.500 and a prevalence of 0.500, pointing to a significant risk.

### Measures of Association for ICIs in Fall Cohorts

This analysis assessed the risk of ICIs across two distinct patient cohorts: Cohort 1, consisting of patients who experienced an initial fall leading to intracranial injury, and Cohort 2, which comprised patients with recurrent falls associated with ICIs. Within Cohort 1, a total of 170 out of 2710 patients (6.273%) sustained ICIs following their initial fall incident. In comparison, Cohort 2 exhibited a higher proportion, with 80 out of 1050 patients (7.619%) affected by ICIs.

Moreover, the calculated risk difference between the two cohorts was −1.346%, with a 95% confidence interval ranging from −3.192% to 0.5%. This negative risk difference implies that the likelihood of ICIs is approximately 1.346% greater for patients experiencing recurrent falls than for those experiencing their initial fall. The associated *z*-score was −1.486, and the p-value was 0.1372, indicating that the observed difference is not statistically significant.

In addition, further analysis utilizing the risk ratio revealed a value of 0.823 (95% confidence interval: 0.637 to 1.064), suggesting that the risk of ICIs is about 17.7% lower in the initial fall cohort compared to the recurrent fall cohort. A risk ratio below 1 reinforces the conclusion that the initial fall cohort has a comparatively lower probability of sustaining ICIs than the recurrent falls cohort.

Furthermore, the odds ratio for ICIs between the two cohorts was calculated at 0.812, with a 95% confidence interval ranging from 0.616 to 1.069. An odds ratio below 1 indicates reduced odds of ICIs in Cohort 1 relative to Cohort 2. Specifically, an odds ratio of 0.812 suggests that the odds of ICIs are approximately 18.8% lower in patients experiencing their initial fall compared to those with recurrent falls.

The Cox proportional hazards model evaluates the relative risks associated with experiencing ICIs after falls, comparing two cohorts. The hazard ratio for patients in Cohort 2 (recurrent falls) compared to those in Cohort 1 (initial falls) is 1.104. This suggests that individuals who have recurrent falls are approximately 10.4% more likely to sustain ICIs than those experiencing their initial fall. The p-value for this finding is 0.0493, indicating statistical significance. The 95% confidence interval for this hazard ratio is 0.833 to 1.463, which is relatively wide but does not include 1, reinforcing the significance of the finding.

Demographic factors, including gender, age, and race, were found to significantly impact the risk of intracranial injury, serving as potential confounders in this analysis. Gender emerged as an important predictor, with males having a hazard ratio of 1.369 compared to females. This suggests that males are 36.9% more likely to experience ICIs following a fall. This association is statistically significant, with a p-value of 0.0186 and a 95% confidence interval of 1.054 to 1.779, indicating that male gender is a notable risk factor for severe injuries and may act as a confounder in the association between other variables and injury risk.

Age also plays a significant role, with each additional year associated with a 2.1% increase in the risk of ICIs (HR = 1.021, *p* = 0.0347). The 95% confidence interval (1.001 to 1.041) excludes 1, supporting the significance of age as a progressive risk factor for ICIs following falls. Age-related physiological changes likely justify this risk increase, and age itself may serve as a confounder that impacts both exposures to falls and the outcome of injury severity.

Race was also analyzed as a factor influencing ICIs risk, though the findings were not statistically significant for all groups. For instance, White individuals had a hazard ratio of 0.735 compared to the reference group, indicating a 26.5% lower risk of intracranial injury. However, this association did not reach statistical significance, with a *p*-value of 0.3474 and a 95% confidence interval of 0.386 to 1.397, which includes 1. This lack of significance suggests that, while race may play a role, the observed association for White individuals may be influenced by other confounding variables or lack sufficient power to establish a definitive link.

Medication use, particularly antihypertensive and antidepressant medications, further influences the risk of ICIs and could serve as a confounding factor due to its impact on both fall risk and injury severity. Individuals taking antihypertensive medications demonstrated a higher risk of sustaining ICIs (HR = 1.958) compared to those not using these medications, suggesting a strong association between use and injury risk, meaning they are 95.8% more likely to sustain ICIs than those not using these medications. This association is highly significant (*p* = 0.0002), with a confidence interval of 1.379 to 2.781, indicating that antihypertensive use substantially increases injury risk. Similarly, antidepressant use is associated with a 47.1% higher risk of ICIs (HR = 1.471, *p* = 0.0093), with a confidence interval of 1.1 to 1.968. These results suggest that the effects of these medications on balance and coordination may contribute to a heightened likelihood of severe injury, further acting as confounders in the relationship between demographic factors and injury outcomes (Table 6).

## DISCUSSION

Falls among older adults have long been recognized as a significant health risk, especially due to their association with severe outcomes like ICIs and the potential for recurrent falls. This study aimed to examine demographic characteristics, risk factors, and injury outcomes associated with initial and recurrent falls resulting in ICIs among older adults aged 65 to 89. By categorizing patients into two cohorts, those who experienced an initial fall and those with recurrent falls within one year, our findings reveal how factors like age, sex, race, and medication use intersect with these risks.

Recurrent falls were associated with a modest but statistically significant increase in ICI risk (HR = 1.104, 95% CI: 0.833–1.463, *p* = 0.0493). Although this hazard ratio reflects borderline significance, the clinical relevance should not be overlooked. In high-risk populations, even small increases in injury risk may translate into considerable burdens on health systems and families, especially when compounded over time.

In examining the relationship between initial and recurrent falls with ICIs, a pattern of increased risk with recurrent falls emerges, even if underlying variables like individual health resilience or comorbid conditions, may affect the observed outcomes. Most gerontology research highlighting how recurrent falls are often accompanied by additional injury risks. This finding reinforces the complex interactions that contribute to fall vulnerability, indicating that factors like functional capacity and overall health play substantial roles [22], even if they do not manifest as large, statistically significant differences in risk levels between cohorts.

Older age remained a consistent and expected predictor of ICI risk. Individuals aged 85 and older demonstrated the highest incidence and prevalence of ICIs, likely due to age-related declines in bone density, muscle strength, and reflexes. These physiological changes contribute to a cumulative trauma effect, where each fall increases vulnerability to subsequent injury. This trend aligns with previous literature suggesting that advanced age amplifies both fall risk and injury severity [23,24].

Men displayed a slightly higher incidence and prevalence of ICIs compared to women, suggesting that gender-based physiological or behavioral factors may play a role. Studies have highlighted that older men might engage in activities that inadvertently increase fall severity, or they might experience falls in ways that result in more impactful injuries [25]. Moreover, male bone density and muscle mass differences compared to females could contribute to a more severe impact when falls do occur, particularly if these falls result in head trauma [26]. This observed gender disparity supports the idea that fall prevention and intervention strategies may benefit from being tailored to address gender-specific risks.

Race presented another intriguing dimension in our analysis, showing variable patterns of fall recurrence and ICI risk among different racial groups. While the majority of individuals experiencing recurrent falls were White, American Indian or Alaska Native participants had the highest ICI incidence and prevalence. Although this finding is limited by the small sample size, it aligns with literature indicating that American Indian and Alaska Native populations often face significant barriers to healthcare access and may have higher rates of comorbidities that exacerbate injury risks [27]. This result is likely influenced by data sparsity and may not reflect a generalizable trend. Additionally, social determinants such as socioeconomic status, healthcare access, and environmental factors likely shape these outcomes, reinforcing the need for culturally sensitive approaches to fall prevention [28]. Future studies with larger, more diverse samples are needed to explore these associations more reliably.

Medication use, particularly antihypertensives (HR = 1.958, *p* = 0.0002) and antidepressants (HR = 1.471, *p* = 0.0093), was strongly associated with ICI risk. These medications can impair balance or reduce blood pressure, increasing the likelihood of falls and injuries, as they can lead to dizziness, unsteadiness, or sudden drops in blood pressure [29,30]. Previous research has underscored the need for careful medication management in fall prevention, especially for older adults already at risk of multiple falls. The results show the importance of balancing medication benefits with potential risks and considering adjustments that minimize fall risk in older adults

Beyond statistical associations, clinical care for older adults who experience falls must consider the balance between treatment efficacy and patient vulnerability. Recent guidelines, such as the 2024 European Society of Cardiology (ESC) recommendations advocating systolic and diastolic targets of 120 to 129 mmHg and 70 to 79 mmHg respectively, may not be suitable for frail older adults [31]. In these patients, overly aggressive blood pressure control can increase fall risk and even mortality [32]. Studies have shown that antihypertensive-induced hypotension, particularly in the presence of aging and comorbidities such as diabetes, which affects more than one third of older adults, may precipitate syncopal events and falls [33]. Evidence suggests that ambulatory blood pressure monitoring for at least 48 to 72 h can help identify periods of hypotension, allowing for more precise adjustment of dosage, timing, or even deprescribing of antihypertensive agents [34]. This individualized approach, as highlighted in recent literature, emphasizes the importance of tailoring pharmacological treatment to blood pressure variability and frailty status rather than applying uniform targets. Such a strategy enhances fall prevention and improves safety for high-risk populations.

Racial differences in fall-related outcomes may also reflect broader disparities in healthcare access, insurance coverage, and the availability of timely preventive or rehabilitative care. Structural factors such as limited access to mobility aids, delayed follow-up, or under-resourced clinical settings may disproportionately affect certain populations, including Black, American Indian, and Alaska Native patients. These systemic inequities should be considered when interpreting racial patterns in fall-related injuries and underscore the need for inclusive, equity-driven prevention strategies.

Future research should include prospective cohort studies to better understand temporal relationships between medication use and intracranial injury risk following falls. In addition, targeted deprescribing interventions in high-risk groups, particularly those with polypharmacy or frailty, may help reduce fall-related complications. Evaluating such strategies in real-world settings could support safer prescribing practices and improve outcomes for vulnerable older adults.

In conclusion, our findings not only affirm trends observed in prior research but also offer refined insights into how recurrent falls impact ICIs risks. By examining the interplay of demographic and physiological factors, our analysis highlights that recurrent falls and cumulative trauma contribute significantly to elevated injury risks among older adults. This reinforces a broader perspective on falls, suggesting they should be viewed as part of a cumulative vulnerability process rather than isolated incidents, with implications for recognizing and mitigating the risks associated with recurrent falls in older populations.

### Study Limitations

This study has several limitations that should be acknowledged. First, the findings may not be applicable outside the Virginia Commonwealth University (VCU) patient population, as the sample is limited to individuals treated within the VCU Health System. Second, using broad ICD codes for ICIs may lead to an underestimation or misclassification of specific injury types, potentially missing patients with unique but unrecognized intracranial conditions. Lastly, our analysis of recurrent falls may be incomplete if patients received care for subsequent falls at outside facilities, potentially resulting in an underreporting of fall recurrence.

### Study Strengths

Despite these limitations, this study is strengthened by its use of real-world data from a large, diverse patient population within a healthcare system, offering valuable insights into fall-related injury patterns and outcomes. This real-world dataset enables a detailed examination of the impact of recurrent falls and provides a practical perspective on fall risks and injury severity in older adults, enhancing the study’s relevance to clinical settings.

## Figures and Tables

**Table 1.** ICD-10 codes for falls and associated ICIs in older adults.

Condition	ICD-10 Code	Description

Fall on same level from slipping, tripping and stumbling	W01.0	Accidental fall due to slipping, tripping, or stumbling
Fall from stairs and steps	W10.9	Unspecified fall from stairs or steps
Fall on and from ladder	W11	Accidental fall from ladder
Fall on and from scaffolding	W12	Accidental fall from scaffolding
Fall on and from building or structure	W13	Accidental fall from a building or structure
Other fall on same level	W18.30	Other falls on the same level
Other fall from one level to another	W17.89	Other falls from one level to another
Fall, unspecified	W19	Unspecified fall
Other specified fall	W18.49	Other specified falls
Fall from furniture	W08	Accidental fall from furniture, such as a bed or chair
Recurrent falls	R29.6	Recurrent falls occurring frequently over time
ICIs with loss of consciousness of 30 min or less	S06.0X1	Concussion with brief loss of consciousness of 30 min or less
ICIs with loss of consciousness from 31 min to 59 min	S06.0X2	Concussion with moderate loss of consciousness from 31 to 59 min
ICIs with loss of consciousness of unspecified duration	S06.0X9	Concussion with unspecified duration of loss of consciousness
ICIs without loss of consciousness	S06.0X0	Concussion without loss of consciousness

**Table 2.** Medication Codes RxNorm and Associated Fall Risk Factors in Older Adults

Condition	RxNorm	Description

Antihypertensive Use	CV490	Other antihypertensives
Antidepressant Use	N06A	General class for antidepressants

**Table 3.** Demographic distribution of older adult patients experiencing initial and recurrent falls (2023–2024).

Variables	Categories	Cohort 1 (Fall) *n* = 2710	Cohort 2 (Recurrent Fall) *n* = 1050	Bassline Comparison	

				*p*-Value	Standard Difference

Age	Current Age (Mean ± SD, Min, Max)	76.1 ± 6.87	76 ± 6.83	0.7023	0.0139
	Age at Index (Mean ± SD, Min, Max)	74.8 ± 6.88	74.6 ± 6.85	0.5622	0.0211
Sex	Female	1580	590	0.2395	0.0427
	Male	1140	470	0.1340	0.0544
	Unknown				
Race	White	1740	710	0.0488	0.0720
	Black or African American	830	290	0.0704	0.0662
	Unknown Race	80	30	0.8769	0.0057
	Other Race	50	20	0.9032	0.0044
	Asian	20	10	0.5074	0.0234
	American Indian or Alaska Native	10	10	0.0274	0.0721
	Native Hawaiian or Other Pacific Islander	0	10	<0.0001	0.1387

**Table 4.** Incidence and prevalence of ICIs following falls in older adults.

Variable	Category	Incidence Proportion	Prevalence	Incidence Rate (Cases/Person-Day)

Age	65–69	0.074	0.153	0.000274
	70–74	0.068	0.127	0.000250
	75–79	0.073	0.133	0.000264
	80–84	0.070	0.133	0.000257
	85 and older	0.125	0.154	0.000477
Sex	Female	0.068	0.127	0.000236
	Male	0.086	0.149	0.000334
	Unknown Gender	0.000	0.000	0.000
Race	American Indian or Alaska Native	1.000	1.000	0.005297
	Asian	0.500	0.500	0.002556
	Black or African American	0.066	0.133	0.000230
	Native Hawaiian or Another Pacific Islander	0.000	0.000	0.000
	Unknown Race	0.125	0.250	0.000583
	White	0.080	0.138	0.000295
	Other Race	0.200	0.200	0.000887

**Table 5.** Incidence and prevalence of recurrent falls and ICIs among older adults.

Variable	Category	Incidence Proportion	Prevalence	Incidence Rate (Cases/PERSON-day)

Age	65–69	0.100	0.217	0.000343
	70–74	0.087	0.160	0.000314
	75–79	0.091	0.167	0.000331
	80–84	0.063	0.118	0.000234
	85 and older	0.222	0.300	0.000922
Sex	Female	0.075	0.153	0.000258
	Male	0.095	0.170	0.000355
	Unknown Gender	0.000	0.000	0.000
Race	American Indian or Alaska Native	0.000	0.000	0.000000
	Asian	0.000	0.000	0.000000
	Black or African American	0.080	0.172	0.000276
	Native Hawaiian or Other Pacific Islander	0.000	0.000	0.000000
	Unknown Race	0.333	0.333	0.001374
	White	0.077	0.155	0.000278
	Other Race	0.500	0.500	0.001656

**Table 6.** Adjusted hazard ratios for ICIs risk factors among older adults following falls.

Covariate	Hazard Ratio (HR)	Coefficient	Standard Error	*z*-Test	*p*-Value	95% Confidence Interval

Age at Index	1.021	0.021	0.01	2.112	0.0347	(1.001, 1.041)
Male	1.369	0.314	0.134	2.354	0.0186	(1.054, 1.779)
Race (Black or African American)	0.705	−0.349	0.159	−2.19	0.0285	(0.516, 0.964)
Antihypertensive Use	1.958	0.672	0.179	3.756	0.0002	(1.379, 2.781)
Antidepressant Use	1.471	0.386	0.149	2.599	0.0093	(1.1, 1.968)

## Data Availability

The data that support the findings of this study are available upon reasonable request. Interested researchers can obtain access to the data by submitting a formal request to the corresponding author at Asmaa.namoos@vcuhealth.org. The data is not publicly available due to privacy or ethical restrictions.
